# Oestrogen receptor-mediated expression of Olfactomedin 4 regulates the progression of endometrial adenocarcinoma

**DOI:** 10.1111/jcmm.12232

**Published:** 2014-02-04

**Authors:** Chao Duan, Xubin Liu, Shuang Liang, Zheng Yang, Meng Xia, Liantang Wang, Shangwu Chen, Li Yu

**Affiliations:** a Department of Pathology, The First Affiliated Hospital, Sun Yat-sen (Zhongshan) UniversityGuangzhou, China; b Gynecology and Obstetrics, The First Affiliated Hospital, Sun Yat-sen (Zhongshan) UniversityGuangzhou, China; c State Key Laboratory for Biocontrol, Guangdong Key Laboratory of Pharmaceutical Functional Genes, Department of Biochemistry, School of Life Sciences, Sun Yat-sen (Zhongshan) UniversityGuangzhou, China

**Keywords:** olfactomedin 4 (OLFM4), endometrial adenocarcinoma, gynecological cancer, oestrogen receptor (ER), endometrial hyperplasia

## Abstract

Endometrial adenocarcinoma is the most common tumour of the female genital tract in developed countries, and oestrogen receptor (ER) signalling plays a pivotal role in its pathogenesis. When we used bioinformatics tools to search for the genes contributing to gynecological cancers, the expression of Olfactomedin 4 (OLFM4) was found by digital differential display to be associated with differentiation of endometrial adenocarcinoma. Aberrant expression of OLFM4 has been primarily reported in tumours of the digestive system. The mechanism of OLFM4 in tumuorigenesis is elusive. We investigated OLFM4 expression in endometrium, analysed the association of OLFM4 with ER signalling in endometrial adenocarcinoma, and examined the roles of OLFM4 in endometrial adenocarcinoma. Expression of OLFM4 was increased during endometrial carcinogenesis, linked to the differentiation of endometrioid adenocarcinoma, and positively related to the expression of oestrogen receptor-α (ERα) and progesterone receptor. Moreover, ERα-mediated signalling regulated expression of OLFM4, and knockdown of OLFM4 enhanced proliferation, migration and invasion of endometrial carcinoma cells. Down-regulation of OLFM4 was associated with decreased cumulative survival rate of patients with endometrioid adenocarcinoma. Our data suggested that impairment of ERα signal-mediated OLFM4 expression promoted the malignant progression of endometrioid adenocarcinoma, which may have significance for the therapy of this carcinoma.

## Introduction

Olfactomedin 4 (OLFM4), designated as human granulocyte colony-stimulating factor-stimulated clone 1 (hGC-1) [1] or GW112 [2], is mainly expressed in the digestive system, prostate and bone marrow. Aberrant expression of OLFM4 has been detected in several types of tumours, especially of the digestive system [3,4]. For example, OLFM4 expression is enhanced in gastric cancer (GC), with 56% of GC cases positive for OLFM4 [3,5]. Overexpression of OLFM4 was found in 75% of head and neck squamous cell carcinomas (HNSCC) validated by immunohistochemistry (IHC), and OLFM4 level is markedly increased in the supernatants of HNSCC cells [6]. Serum OLFM4 concentration in patients with GC and pancreatic cancer is significantly higher than that in healthy individuals [5,7]. Increased expression of OLFM4 was observed in peripheral blood mononuclear cells of pancreatic adenocarcinoma patients [8]. Its level is also associated with differentiation and malignant progression of gastro-enteric tumours. Expression of OLFM4 is low or absent in poorly differentiated or undifferentiated gastric and colon cancers and enhanced in more differentiated cancers [3,4]. Olfactomedin 4 expression is down-regulated in advanced gastric and colon cancers [4,5]. Olfactomedin 4 has therefore been identified as a tumour marker or a potential prognostic marker [9–11]. In addition, OLFM4 may be a marker for stem cells in human intestine and for a subset of colorectal cancer (CRC) cells [12,13]. It is believed that the role of OLFM4 in tumourigenesis involves its effects on apoptosis and the cell cycle. Forced expression of OLFM4 in cancer cells has been reported to show anti-apoptotic action and promote tumour growth *in vivo* [2]. Olfactomedin 4 expression is elevated during the early S phase of the cell cycle, and knockdown of OLFM4 by siRNA increases S phase cell populations, resulting in a cell cycle arrest at the S phase [14].

Endometrial adenocarcinoma is the most common malignant tumour of the female genital tract in developed countries, ranking fourth in incidence among invasive tumours in women. It is estimated that 47,130 new endometrial cancer cases and 8010 deaths occurred in the United States in 2012 [15]. Over 80% of endometrial cancers are typical adenocarcinomas, also known as endometrioid adenocarcinomas. Despite its high incidence, the molecular events that contribute to the development and progression of the lesion remain poorly understood. Mutations of several molecules, including PTEN, KRAS, Her/neu-2 and P53 [16,17], have been reported to contribute to endometrial carcinogenesis, and some of these act as oncogenic factors [18,19].

Studies of OLFM4 in tumourigenesis have mainly focused on gastrointestinal tumours, but the role of OLFM4 in other tumours is not well-known. Recent research has found OLFM4 to be highly expressed in proliferative-phase endometrium, and OLFM4 expression is associated with the presence of epidermal growth factor receptor-1 and oestrogen receptor-α (ERα) in endometriosis and endometrial cancer [20]. This study also showed OLFM4 transcription to be up-regulated in the endometrium of patients with endometriosis [20]. In an earlier study, we found that the OLFM4 level was elevated with increased pathological grade of pre-cancerous cervical squamous intraepithelial lesions [21]. Olfactomedin 4 expression was higher in well-differentiated invasive squamous cell carcinomas (ISCCs) than in poorly differentiated ISCCs. This suggested that OLFM4 was associated with progression of cervical intraepithelial neoplasia and differentiation of cervical cancer, playing an important role in tumours of the female reproductive tract. When we searched for genes contributing to gynecological cancers through bioinformatics tools, we found OLFM4 to be associated with differentiation of endometrial adenocarcinoma. In the present study, we investigated OLFM4 alteration during endometrial carcinogenesis, analysed the correlation of OLFM4 expression with oestrogen receptor signalling and regulation of OLFM4 expression in endometrial adenocarcinoma, and examined the effects of OLFM4 on biological features of endometrial carcinoma cells and progression of endometrial adenocarcinoma.

## Materials and methods

### Databases and digital differential display

*In silico* analysis was based on NCBI EST (http://www.ncbi.nlm.nih.gov) and UniGene databases (http://www.ncbi.nlm.nih.gov/UniGene). UniGene is an analytical system for producing an organized view of the transcriptome. It employs a computer program to assign human expressed sequence tags (EST) sequences to distinct clusters. Each cluster, including a set of transcript sequences, represents a unique human expressed gene, and is assigned a UniGene entry number.

Analysis of genes differentially expressed in well-, moderately- and poorly-differentiated endometrial adenocarcinomas was performed using a web-based digital differential display (DDD). This is a data-mining bioinformatics tool available to researchers through the NCBI website [22,23]. It compares the EST-based expression profiles of two or more libraries, or library pools, in the UniGene database and allows the identification of genes with significantly different expression levels (differentially expressed genes) among libraries, making it possible to identify specific genes that may contribute to a particular cell characteristic such as differentiation or tumourigenesis [23,24]. Digital differential display uses Fisher*s exact test to restrict the output to significant differences. The analysis is also restricted to deeply sequenced libraries, and only those with over 1000 sequences in UniGene are included in DDD [23].

Three libraries of endometrial adenocarcinoma at various differentiation levels (Table S1) were selected from the UniGene database for DDD analysis. The libraries involved in the study were similar in all aspects with the exception of the differentiation levels.

### Digital expression analysis of differentially expressed genes

*In silico* tissue-specificity profiles of differentially expressed genes identified with DDD were obtained *via* the web interfaces of UniGene*s EST ProfileViewer (http://www.ncbi.nlm.nih.gov/UniGene), which is based on the analysis of EST counts in the pool of libraries. The expression level of each gene was defined as the number of a specific gene EST/total EST in the pool. Seventy pools of libraries derived from different anatomical sites and corresponding tumour tissues were examined.

### Subjects and tissue samples

Formalin-fixed, paraffin-embedded (FFPE) tissues of primary endometrioid adenocarcinomas (*n* = 200; mean patient age = 53.3) and corresponding patient clinicopathological data were collected from January 1998 to December 2010 at the Department of Pathology, First Affiliated Hospital of Sun Yet-sen University. Patients were followed up until 1 June 2012 or until death. None had received pre-operative radiotherapy, chemotherapy or hormone drug therapy. Patients were evaluated in accordance with the International Federation of Gynecology and Obstetrics (FIGO) criteria 2009. The number of cases classified as FIGO stage I, II, III and IV were 147, 17, 30 and 6, respectively. Histological grade was determined in accordance with the World Health Organization (WHO) criteria 2003. The number of cases classified as WHO grade 1, 2 and 3 were 86, 84 and 30, respectively. Formalin-fixed, paraffin-embedded specimens of normal endometrium (*n* = 30), endometrial hyperplasia (*n* = 30) and endometrial hyperplasia with atypia (*n* = 30) were also collected. Formalin-fixed, paraffin-embedded tissues were sectioned and stained with haematoxylin-eosin or immunostained with OLFM4, ERα and progesterone receptor (PR) antibodies. Fresh tissue specimens of normal endometrium (*n* = 19), endometrial hyperplasia (*n* = 20) and primary endometrioid adenocarcinoma (*n* = 32; mean patient age = 59.0) were collected from women undergoing hysterectomy June 2011 to October 2012 at the First Affiliated Hospital of Sun Yet-sen University. The number of cases classified as WHO grade 1, 2 and 3 were 9, 17 and 6, respectively. All research involving human subjects was approved by the Medical Ethics Review Board of the First Affiliated Hospital, Sun Yat-sen University, in accordance with the guidelines for the protection of human subjects. Written informed consent was obtained from each participant/guardian.

### Steroid hormones and chemicals

17β-estradiol (E_2_) was purchased from Sigma-Aldrich (St. Louis, MO, USA). 4′,4″,4‴-(4-propyl-[1H]-pyrazole-1,3,5-triyl)trisphenol (PPT), 2, 3-bis-(4–ydroxyphenyl)–propionitrile (DPN) and ICI 182 780 were purchased from Tocris Cookson Ltd (Bristol, UK).

### Immunohistochemistry

Sections (4 μm) were cut from FFPE tissue blocks, and IHC was conducted as described previously [25] with antibodies to OLFM4 (ab96280, rabbit anti-human polyclonal, diluted at 1/100; Abcam, Cambridge, England), ERα (Kit-0012-2, rabbit anti-human monoclonal, ready to use; Maixin Biotechnology, Fuzhou, Fujian, China), PR (Kit-0013-2, rabbit anti-human monoclonal, ready to use; Maixin Biotechnology), and horseradish peroxidase-labelled secondary antibody (Maixin Biotechnology) in accordance with manufacturer*s instructions. Colour was developed with diaminobenzidine (Dako Corp, Carpinteria, CA, USA) incubated for 5–10 min. at room temperature. Slides were counterstained with haematoxylin and examined by light microscopy.

The immunoreactive score (IRS) was used to evaluate results: IRS = staining intensity × percent of positive cells. Staining intensity was graded according to the following criteria: 0 (no staining), 1 (weak staining, light yellow), 2 (moderate staining, yellow with brown) and 3 (strong staining, brown). The percent staining was graded according to the proportion of positive stained cells as follows: 0 for ≤5% positive cells; 1 for 6–25% positive cells; 2 for 26–50% positive cells and 3 for ≥51% positive cells. An IRS score of 4 and higher is regarded as high expression of genes.

### Cell culture and treatment

Ishikawa, a well-differentiated adenocarcinoma cell line, was purchased from Guangzhou Qiyun Biotechnology Co. Ltd. (Guangzhou, China), and HEC-1B, a moderately-differentiated adenocarcinoma cell line, was obtained from the Cell Bank of the Chinese Academy of Sciences (Shanghai, China). The cells were maintained in RPMI 1640 medium (Life Technologies, Carlsbad, CA, USA) supplemented with 10% foetal bovine serum (Invitrogen, Carlsbad, CA, USA). For the knockdown experiment, Ishikawa cells were seeded in 6-well plates for 24 hrs before transfection. Cells grown to 30–50% confluence were transfected using Lipofectamine™2000 (Invitrogen) with small interfering RNA (siRNA) duplexes specific for human ERα, OLFM4, or negative control siRNA according to the manufacturer*s protocol. Initially, four sets of siRNA duplexes of differing concentrations were tested to evaluate the target specificity and efficacy. The siRNA duplexes showing the most efficient knockdown of ERα and OLFM4 were used for further experiments at 50 nmol/l concentration. For OLFM4 over-expression, the OLFM4 gene was cloned into GV166 (Ubi-MCS-3FLAG-IRES-puromycin) lentiviral vector (Shanghai Genechem Co. Ltd.). The construct was used to transfect HEC-1B cells. GV166-null was used as a control virus.

### Quantitative real-time reverse transcription PCR

Total RNA was extracted from cultured cells or fresh endometrial tissue using Trizol reagent (Invitrogen Life Technology) according to the manufacturer*s protocol, and 800 ng of total RNA was converted to cDNA with a First Strand cDNA Synthesis Kit (Toyobo, Osaka, Japan). The primers for OLFM4, ERα, PR and GAPDH were synthesized by Shanghai Sangon Company (Table S2). Reactions were conducted in a 20 μl reaction volume in triplicate using FastStart Universal SYBR Master (Rox; Roche, Mannheim, Germany) and Applied Biosystems 7500 Real-Time PCR System. Expression fold-change of genes was evaluated using 2^−ΔΔCt^.

### Western blotting

Nuclear or plasma protein was isolated from cells with a nucleoprotein and cytoplasm protein extraction kit (Keygen, Nanjing, China), and total protein was isolated with a total protein extraction kit (Keygen). Concentration of protein was determined with the BCA protein assay kit (Cowin BioTech, Beijing, China). Proteins (40 μg) were resolved by SDS–PAGE, transferred to a PVDF membrane, and probed with antibodies to ERα (sc-71064, 1:400; Santa Cruz Biotechnology, Dallas, TX, USA), Histone H3 (bs-0349R, 1:200; Bioss, Woburn, MA, USA), and ERβ and PR-A/B(#5513 and #3176S, 1:1000; Cell Signalling Biotechnology, Danvers, MA, USA). Proteins were visualized using horseradish peroxidase-conjugated secondary antibody, followed by development with ECL reagents (Amersham, Little Chalfont, Buckinghamshire, UK).

### Cell proliferation assay

Cell proliferation was detected using 3-(4,5-dimethylthiazol-2-yl)-2,5-diphenyltetrazolium bromide (MTT) (Sigma-Aldrich). Cells in logarithmic phase were seeded at 3 × 10^3^ per well in 96-well plates. Cells grown to 30–50% confluence were transfected with siRNA specific for OLFM4 or negative control siRNA. After 24 hrs, cells were treated with E_2_ at 10^−9^ mol/l for 72 hrs. At the end of the treatment, MTT reagent was added to the medium at a concentration of 1 mg/ml and incubated at 37°C for an additional 4 hrs. The reaction was terminated with 150 μl dimethylsulphoxide per well. The plates were gently shaken for 15 min. Absorbance values were measured using a microplate reader at 490 nm, and the results were plotted as mean ± SD.

### Wound healing assay

The capacity for cell migration was determined by a wound healing assay. Confluent cells were starved for 6 hrs in serum-free medium and wounded by scratching with a 20 μl micropipette tip for analysis of wound closure. Cells were washed three times with PBS and cultured for 48 hrs in serum-free medium. The width of wound area was monitored at 0, 6, 12, 24 and 48 hrs and photographed through an inverted microscope.

### Transwell cell migration and invasion assay

For transwell cell migration and invasion assay, 2 × 10^5^ cells were plated in the upper chamber, and 500 μl medium with 10% foetal bovine serum was added to the lower chamber of the transwell. The transwell was incubated at 37°C in a 24-well plate for 48 hrs. Non-invasive cells on the surface of the upper chamber were gently wiped away with a cotton-tipped swab, and the cells under the surface of the lower chamber were fixed in 4% paraformaldehyde and stained with 0.1% crystal violet for 20 min. at room temperature. For transwell invasion assay, the upper chamber was coated with 50 μl Matrigel (1 mg/ml; BD Biosciences, San Jose, CA, USA) and held at 37°C for 24 hrs to solidify the Matrigel.

### Cell apoptosis assay

Flow Cytometry was used for the cell apoptosis assay with a fluorescent Annexin V-FITC assay kit (Keygen). 3 × 10^5^ cells were pelleted by centrifugation, washed twice with ice-cold PBS, and resuspended in Annexin V-FITC reagent in the dark for 15 min. A FACS Calibur flow cytometer (BD Biosciences) was used for flow cytometric analysis.

### Statistical analysis

SPSS 16.0 software (SPSS, Inc., Chicago, IL, USA) was used for data analysis. Statistical comparisons of quantitative data were made using Student*s *t*-test or anova. The expression differences of OLFM4, ERα and PR among multiple groups were analysed by a chi-squared test, Fisher*s exact test, or the Wilcoxon rank sum test. Associations between OLFM4 expression and ERα or PR were analysed by Pearson chi-squared test or Spearman rank correlation test. The chi-squared test was also used to analyse the relationships between OLFM4 expression and clinico-pathological characteristics. The Kaplan–Meier method was used to calculate the survival curve. A log-rank test was used for univariate survival analysis. A *P* < 0.05 was considered statistically significant. When comparing two groups, the Bonferroni method was used to adjust the inspection standard.

## Results

### OLFM4 was identified by DDD as a differentially expressed gene associated with differentiation of endometrial adenocarcinoma

To explore the genes that might contribute to tumour differentiation, three UniGene libraries derived respectively from well-, moderately- and poorly-differentiated endometrial adenocarcinomas were subjected to DDD analysis. Several genes displayed statistically significant differences in EST counts (data not shown). Of these genes, OLFM4 was markedly down-regulated in poorly-differentiated endometrial adenocarcinoma compared with well-differentiated.

To determine the distribution of OLFM4 in various tissues including tumours, OLFM4 expression level was viewed using UniGene*s EST ProfileViewer. As previously reported [3–5], OLFM4 is highly represented in several library pools including colorectal tumour, bladder and intestine (Table S3). The expression of OLFM4 in uterine tumour was 2.4-fold that in uterus, indicating that OLFM4 transcription is up-regulated in uterine tumours.

### Expression of OLFM4 was increased during endometrial carcinogenesis

**Fig. 1:**
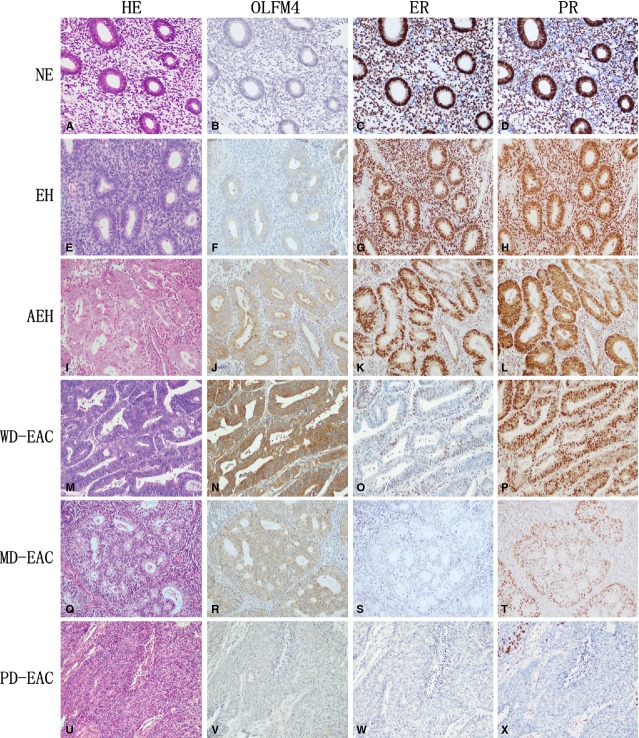
Expression of Olfactomedin 4 (OLFM4), estrogen receptor-α (ERα) and progesterone receptor (PR) in normal endometrium, endometrial hyperplasia and endometrioid adenocarcinoma detected by immunohistochemistry. Haematoxylin-eosin, Morphology of normal endometrium (**A**), simple hyperplasia without atypia (**E**), complex atypical hyperplasia (**I**), and well-(**M**), moderately-(**Q**) and poorly-differentiated endometrioid adenocarcinomas (**U**) stained by haematoxylin-eosin. OLFM4, OLFM4 staining was undetectable in normal endometrium (**B**). Immunoreactivity of OLFM4 gradually increased from endometrial hyperplasia (**F**), atypical endometrial hyperplasia (**J**) to well-differentiated endometrioid adenocarcinoma (**N**), and gradually decreased with lower degrees of differentiation in endometrioid adenocarcinomas (**N**, **R**, **V**). ER, Immunoreactivity of ER gradually decreased from normal endometrium (**C**), endometrial hyperplasia (**G**), atypical endometrial hyperplasia (**K**) to well-differentiated endometrioid adenocarcinoma (**O**). ER staining was hardly detectable in moderately-(**S**) and poorly-(**W**) differentiated endometrioid adenocarcinomas. PR, Immunoreactivity of PR gradually decreased from normal endometrium (**D**), endometrial hyperplasia (**H**), atypical endometrial hyperplasia (**L**), well- (**P**), moderately-(**T**) to poorly-(**X**) differentiated endometrioid adenocarcinomas. NE: normal endometrium; EH: endometrial hyperplasia; AEH: atypical endometrial hyperplasia; WD-EAC: well-differentiated endometrioid adenocarcinoma; MD-EAC: moderately-differentiated endometrioid adenocarcinoma; PD-EAC: poorly-differentiated endometrioid adenocarcinomas.

**Fig. 2:**
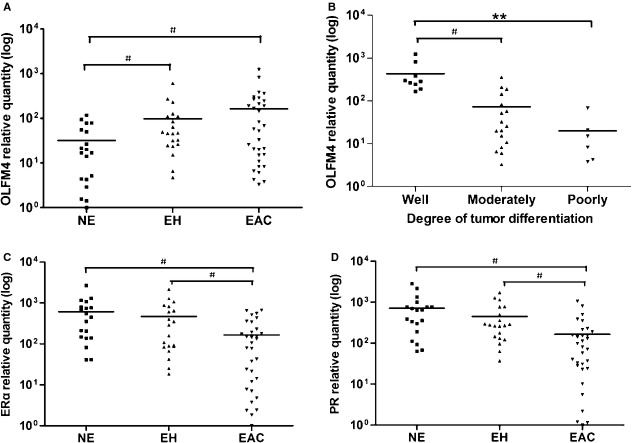
Olfactomedin 4 (OLFM4), oestrogen receptor-α (ERα) and progesterone receptor (PR) mRNA level in endometrial tissues measured by real-time RT-PCR. (**A**) OLFM4 mRNA level in normal endometrium, endometrial hyperplasia and endometrioid adenocarcinoma. (**B**) OLFM4 mRNA level in well-, moderately- and poorly-differentiated endometrioid adenocarcinomas. (**C**) ERα mRNA level in normal endometrium, endometrial hyperplasia and endometrioid adenocarcinoma. (**D**) PR mRNA level in normal endometrium, endometrial hyperplasia and endometrioid adenocarcinoma. For multiple comparisons between groups the significance level was adjusted to 0.05/3 = 0.017. ^#^*P* < 0.017, **P < 0.001. NE: normal endometrium; EH: endometrial hyperplasia; EAC: endometrioid adenocarcinoma.

To confirm the *in silico* analysis, we assessed the expression of OLFM4 in normal endometrium, precancerous endometrial tissue and endometrial cancer using IHC. Olfactomedin 4 proteins were stained in the cytoplasm of glandular cells (Fig. 1). The rates of OLFM4 high-expression in normal endometrium, endometrial hyperplasia without or with atypia, and endometrioid adenocarcinoma were 43.3, 60.0, 66.7 and 68.0%, respectively (Table S4). The OLFM4 expression level in endometrioid adenocarcinoma was significantly higher than in normal endometrium (*P* = 0.008), indicating that OLFM4 expression is up-regulated in endometrial carcinogenesis. Similar to what was observed with IHC, the OLFM4 mRNA level detected by real-time PCR in endometrial hyperplasia and endometrioid adenocarcinomas was higher than that in normal endometrium (*P* < 0.017, Fig. 2A).

### The expression of OLFM4 was linked to the differentiation of endometrial adenocarcinoma

To investigate whether OLFM4 expression is associated with tumour differentiation, the relationship of OLFM4 level to differentiation level of endometrial adenocarcinoma was analysed. Immunostaining intensity of OLFM4 gradually decreased as the degree of differentiation declined in endometrioid adenocarcinomas (Fig. 1N, R and V). High-expression rates detected by IHC in well-, moderately- and poorly-differentiated endometrioid adenocarcinomas were 81.4, 65.5, and 36.7%, respectively. The differences are statistically significant (*P* < 0.001, Table S5). This tendency was also observed in OLFM4 mRNA levels analysed by real-time PCR (Fig. 2B). Olfactomedin 4 mRNA in Ishikawa, a well-differentiated human endometrial carcinoma cell line, was also higher than that in moderately-differentiated HEC-1B cells (Fig. S1B and C). The results suggested that OLFM4 expression was lower in less differentiated endometrial adenocarcinoma.

### Estrogen receptor signalling regulated expression of OLFM4

A previous study demonstrated that oestrogen receptor signalling regulated the expression of OLFM4 in uterine endometrium [20]. We first investigated the expression of ER and PR in endometrioid adenocarcinoma. Marked expression of ERα was detectable with IHC in 96.7% of specimens of normal endometrial tissue, 93.3% of endometrial hyperplasia samples, 73.3% of atypical endometrial hyperplasia samples, and 45.5% of endometrioid adenocarcinoma samples (*P* < 0.001, Table S4, Fig. 1). High-expression rates of PR in these tissues were 96.7, 96.7, 83.3 and 66.5%, respectively (*P* = 0.001, Table S4, Fig. 1). The expression of ERα and PR was down-regulated with lower degrees of differentiation in endometrioid adenocarcinomas (Fig. 1). Detection of ERα and PR mRNA with real-time PCR also showed the expression of receptors to gradually decrease when normal endometrium developed into endometrial carcinoma (Fig. 2C and D). Expression of OLFM4 in endometrioid adenocarcinoma was positively correlated with the expression of ERα and PR (Table 1 and Fig. S2).

**Table 1:** Co-relationship of OLFM4 level with ERα and PR expression in endometrial tissues based on IHC data

	Endometrium (*n* = 30)		Hyperplasia (*n* = 30)		Atypical hyperplasia (*n* = 30)		Adenocarcinoma (*n* = 200)	
				
OLFM4	High-exp	Low-exp	High-exp	Low-exp	High-exp	Low-exp	High-exp	Low-exp
ERα								
High-exp	13	0	18	0	15	5	79	57
Low-exp	16	1	10	2	7	3	12	52
*P*	0.374		0.073		0.770		<0.001	
*r*	–		–		–		0.369	
PR								
High-exp	13	0	18	0	17	3	101	35
Low-exp	16	1	11	1	8	2	32	32
*P*	0.374		0.213		0.729		0.001	
*r*	–		–		–		0.240	

**Fig. 3:**
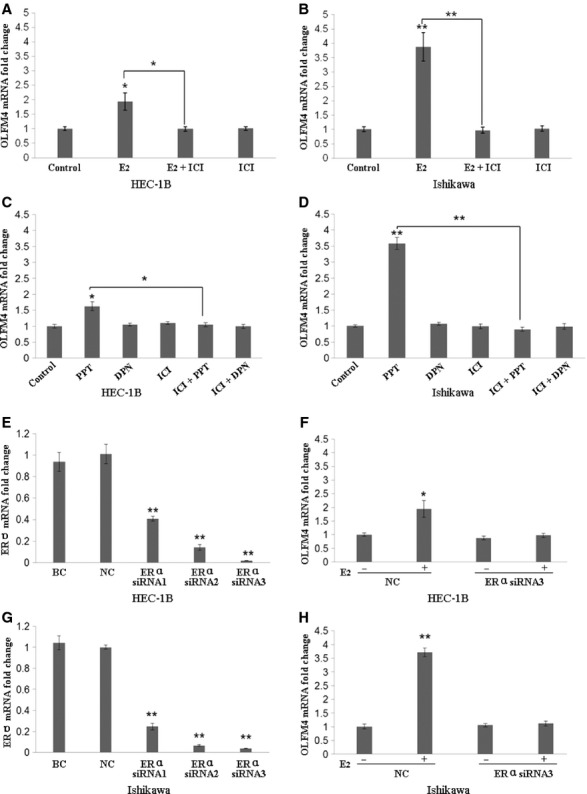
Expression of Olfactomedin 4 (OLFM4) is regulated by the oestrogen receptor-α (ERα) signalling in endometrial carcinoma cells. (**A** and **B**) Oestrogen antagonist ICI 182 780 attenuated the OLFM4 mRNA increase induced by E_2_. (**C** and **D**) ERα-specific agonist PPT, but not ERβ-specific agonist DPN, induced OLFM4 mRNA expression. (**E**–**H**) Knockdown of ERα with siRNA reduced E_2_-induced expression of OLFM4. *P < 0.05, **P < 0.001. E_2_, 17β-estradiol; ICI, ICI 182 780; PPT, 4′,4″,4‴-(4- propyl-[1H]-pyrazole-1,3,5-triyl)trisphenol; DPN, 2,3-bis-(4–ydroxyphenyl)–propionitrile; BC: blank control; NC: negative control (non-specific siRNA group).

We also investigated whether the expression of OLFM4 in endometrial adenocarcinoma cell lines is regulated by oestrogen receptor signalling. Both Ishikawa and HEC-1B cells expressed ERα, ERβ, PR-A and PR-B, but the expression levels in Ishikawa cells were higher than that in HEC-1B (Fig. S1A). Similarly, OLFM4 mRNA expression in HEC-1B cells was lower than that in Ishikawa cells (Fig. S1B and C). When the cells were stimulated with oestrogen E_2_, OLFM4 mRNA expression was increased (Fig. 3A and B). Addition of oestrogen receptor antagonist ICI 182 780 attenuated the OLFM4 mRNA increase induced by E_2_. In contrast, OLFM4 mRNA expression was enhanced upon the stimulation of ERα-specific agonist PPT, whereas ERβ-specific agonist DPN had no effect (Fig. 3C and D). ICI 182 780 attenuated the OLFM4 mRNA increase induced by PPT. Knockdown of ERα in HEC-1B and Ishikawa cells using siRNA attenuated the E_2_-induced expression of OLFM4 (Fig. 3F and H). Results suggested that ERα-mediated signalling regulated expression of OLFM4.

### OLFM4 suppressed proliferation, metastasis and invasion of endometrial adenocarcinoma cells

**Fig. 4:**
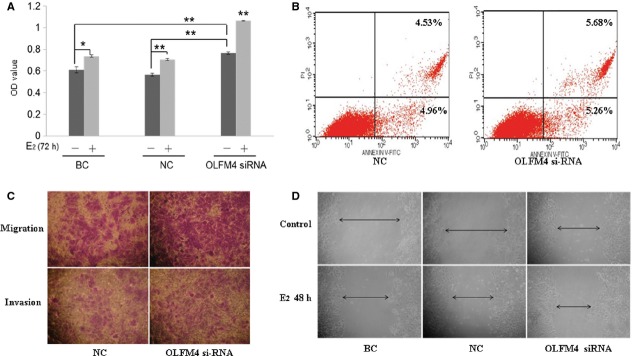
Effects of Olfactomedin 4 (OLFM4) on the biological features of endometrial carcinoma cells. (**A**) Effects of OLFM4 on proliferation of Ishikawa measured by MTT (*P < 0.05, **P < 0.001). (**B**) OLFM4 did not affect apoptosis in Ishikawa cells analysed by flow cytometry. (**C**) Effects of OLFM4 on migration and invasion of Ishikawa cells measured by transwell assay (200×). (**D**) *In vitro* scratch assay detected effects of OLFM4 on Ishikawa cell migration (100×).

We further studied the effects of OLFM4 on biological features of endometrial adenocarcinoma cells. E_2_-treatment slightly enhanced the proliferation of Ishikawa cells (Fig. 4A). Knockdown of OLFM4 increased tumour cell proliferation with and without E_2_ stimulation, suggesting that OLFM4 partially suppresses tumour cell proliferation. However, reducing OLFM4 expression did not affect cell apoptosis (Fig. 4B). The *in vitro* scratch assay demonstrated that suppression of OLFM4 expression with siRNA increased Ishikawa cell migration (Fig. 4D). The transwell migration and invasion assay indicated that knockdown of OLFM4 enhanced the tumour cell capacity for migration and invasion (Fig. 4C). In contrast, OLFM4 over-expression suppressed proliferation and invasion of endometrial adenocarcinoma cells (data not shown). It appeared that OLFM4 was involved in suppression of migration and invasion of endometrial carcinoma cells.

### OLFM4 expression was associated with the prognosis of endometrioid adenocarcinoma

**Fig. 5:**
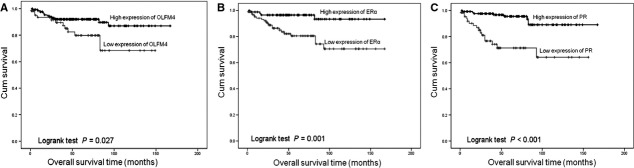
Cumulative survival curve of patients with endometrioid adenocarcinoma and expression levels of Olfactomedin 4 (**A**), oestrogen receptor-α (**B**) and progesterone receptor (**C**).

Based on the expression traits of OLFM4 measured by IHC, we analysed the association of OLFM4 level with clinico-pathological features and prognosis of endometrioid adenocarcinoma. Olfactomedin 4 expression was significantly associated with patient age, tumour FIGO stage, histological grade and myometrial invasion and not significantly associated with menostasia or lymph node metastasis (Table 2). Of the 200 followed up cases, 24 patients died. The cumulative survival rate was 88.0%. Cumulative survival rate of 136 cases with high-expression of OLFM4 (91.2%) and 64 cases with low-expression of OLFM4 (81.2%) was significantly different (χ^2^ = 4.879, *P* = 0.027, Fig. 5). Both ERα and PR expression was significantly associated with FIGO stage, histological grade, myometrial invasion and lymph node metastasis. Progesterone receptor expression was also associated with patient age and menostasia. Cumulative survival rate in cases with high- and low-expression of ERα was 95.6% and 81.7%, respectively, and with high- and low-expression of PR was 94.0% and 76.1%, respectively. The differences in cumulative survival rate between high-expression and low-expression groups were significant (Fig. 5). The data suggested that patients with high-expression of OLFM4 and those with high ERα and PR have a better prognosis compared with low-expression cases.

**Table 2:** Association of OLFM4, ERα and PR expression detected by IHC with clinico-pathological features of endometrioid adenocarcinoma

	OLFM4 expression			ERα expression			PR expression		
			
Parameters	High	Low	*P*	High	Low	*P*	High	Low	*P*
Age									
<60 (*n* = 140)	103	37	0.010	70	70	0.051	102	38	0.004
≥60 (*n* = 60)	33	27		21	39		31	29	
Menostasia									
No (*n* = 91)	66	25	0.210	47	44	0.111	69	22	0.011
Yes (*n* = 109)	70	39		44	65		64	45	
FIGO stages									
I (*n* = 147)	107	40	0.016	75	72	0.009	109	38	<0.001
II–IV (*n* = 53)	29	24		16	37		24	29	
Histological grade									
Well (*n* = 86)	70	16	<0.001	63	23	<0.001	73	13	<0.001
Moderate(*n* = 84)	55	29		25	59		52	32	
Poor (*n* = 30)	11	19		3	27		8	22	
Myometrial invasion									
<1/2 (*n* = 143)	105	38	0.009	74	69	0.005	102	41	0.022
≥1/2 (*n* = 57)	31	26		17	40		31	26	
Lymph node metastasis									
No (*n* = 182)	125	57	0.511	88	94	0.010	129	53	<0.001
Yes (*n* = 18)	11	7		3	15		4	14	

## Discussion

Olfactomedin 4 has been identified as a molecule involved in development and progress of gastrointestinal cancers. Its effect on gynecological tumors has not been well-studied. In the present study, intensive immunoreactivity for OLFM4 was observed in endometrioid adenocarcinoma, and its expression was linked to the level of differentiation of the tumour. Expression of OLFM4 in endometrioid adenocarcinoma was positively related to expression of ERα and PR. *In vitro* studies demonstrated that ERα-mediated signalling regulated expression of OLFM4, and, in turn, OLFM4 suppressed proliferation, migration and invasion of endometrial carcinoma cells. Low-expression of OLFM4 was associated with reduced cumulative survival rate of patients with endometrioid adenocarcinoma.

Association of OLFM4 with tumour differentiation has been reported in gastric and colon cancers. Olfactomedin 4 expression was highly up-regulated in more differentiated cancers and remarkably reduced or absent in poorly differentiated or undifferentiated cancers [3,4]. In an earlier study, preliminary data suggested that low-expression of OLFM4 correlated with poor differentiation of endometrial adenocarcinoma [26]. The current study further supported a contribution of OLFM4 to differentiation of endometrial carcinoma.

The mechanism of OLFM4 in tumourigenesis is elusive. In general, the role of OLFM4 may lie in its capability of regulating the cell cycle and apoptosis as well as cell adhesion and migration [2,14,27,31]. It has been reported that OLFM4 possesses anti-apoptotic properties counteracting H_2_O_2_- or other cytotoxic agent-induced apoptosis and promotes proliferation of cancer cells [2,20,27]. Anti-apoptotic activity of OLFM4 may rely on its association with a potent apoptotic inducer, GRIM-19. Enhancement of transition from the S to G2/M phase in pancreatic cancer cells was shown to potentiate cell proliferation [14]. However, these results are controversial. It was found that OLFM4 significantly suppressed the tumourigenicity of mouse melanoma cell B16F10, but had no positive effect on cell viability or cell cycle progression [28]. Olfactomedin 4 suppressed prostate cancer cell growth and metastasis *via* interaction with cathepsin D and stromal cell derived factor-1-1 [29]. Olfactomedin 4 over-expression in leukaemia cells led to growth inhibition, differentiation and apoptosis [30]. Olfactomedin 4 knockdown did not trigger obvious cell apoptosis in GC cells but increased H_2_O_2_- and tumour necrosis factor-α-induced apoptosis [7]. Our results demonstrated that OLFM4 itself did not affect the apoptosis of endometrial carcinoma cells, but it may slightly inhibit cell proliferation.

It has been suggested that OLFM4 may facilitate cell adhesion through an interaction with endogenous cell surface lectins and cadherin [31]. However, OLFM4 over-expression was shown to decrease cell adhesion and migration but not to alter proliferation of human colon carcinoma (HT-29) cells [4]. Forced expression of OLFM4 in HEK-293 cells resulted in decreased vimentin expression and decreased cell adherence [20]. Olfactomedin 4 suppressed the migration and invasion of mouse melanoma cells through down-regulation of integrin and matrix metalloproteinase expression [28]. Our results showing OLFM4 to suppress migration and invasion by endometrial carcinoma cells support the suggestion that OLFM4 may function as a tumour suppressor during tumour progression.

Olfactomedin 4 immunostaining frequency or expression level is decreased in advanced gastric and colon cancers [4,5,32], indicating that OLFM4 is a valuable marker for prognosis of gastrointestinal tumours. Patients with OLFM4-positive CRC showed a better survival rate than patients with OLFM4-negative CRC [32]. Down-regulation of OLFM4 in GC is significantly associated with lymph node and distant metastases and with poor prognosis [33]. Our results, indicating that down-regulation of OLFM4 reduced the cumulative survival rate of patients with endometrioid adenocarcinoma, were consistent with these findings. However, it has been reported that, in pancreatic ductal adenocarcinomas, OLFM4 expression was up-regulated in the poor-prognosis group, and high expression of OLFM4 correlated significantly with lower overall survival [34].

Oestrogen receptor signalling plays a critical role in pathogenesis of endometrial carcinoma, but the underlying mechanism is not clear. Elucidation of the effect of OLFM4 on gynecological tumour and its association with ER signalling is of great importance. The immunostaining of OLFM4 in endometrial cancer has been observed to be correlated with ERα staining [20]. However, the expression levels of OLFM4 in endometrial carcinomas were found comparable to those in healthy postmenopausal endometrium, although an increase of OLFM4 mRNA was observed in the eutopic endometrium of women with endometriosis compared with controls [20]. Since only nine patients were engaged in this study, the validity of the conclusions may be questioned [20]. In an earlier study, we found that OLFM4 expression was up-regulated in cervical epithelial carcinogenesis and linked to differentiation of cervical cancer, indicating that OLFM4 plays a role in gynecological cancer [21]. In the current study, we further demonstrated that OLFM4 expression correlated with the differentiation of endometrial adenocarcinoma, and dysfunction of ER signalling-mediated OLFM4 expression promoted the malignant progression of the cancer, which may potentially have significance for the treatment of endometrial adenocarcinoma.

## Supporting information

**Figure S1**. Expression of OLFM4, ER and PR in endometrial carcinoma cells.

**Figure S2**. Co-relationships of OLFM4 with ERα or PR in mRNA level in normal endometrium and endometrial lesions.

**Table S1**. UniGene libraries involved in digital differential display.

**Table S2**. Primers used for PCR analysis in the study.

**Table S3**. Expression profile of OLFM4 analysed by EST count.

**Table S4**. Expression of OLFM4, ERα and PR in endometrial tissues detected by IHC.

**Table S5**. Differentiation of endometrioid adenocarcinoma and OLFM4 expression level.
