# Supplementary figures and images for: Oestrogen receptor-mediated expression of Olfactomedin 4 regulates the progression of endometrial adenocarcinoma
Source: J Cell Mol Med. 2014 Feb 4;18(5):863–74. doi: 10.1111/jcmm.12232 (PMC4119392; doi:10.1111/jcmm.12232)

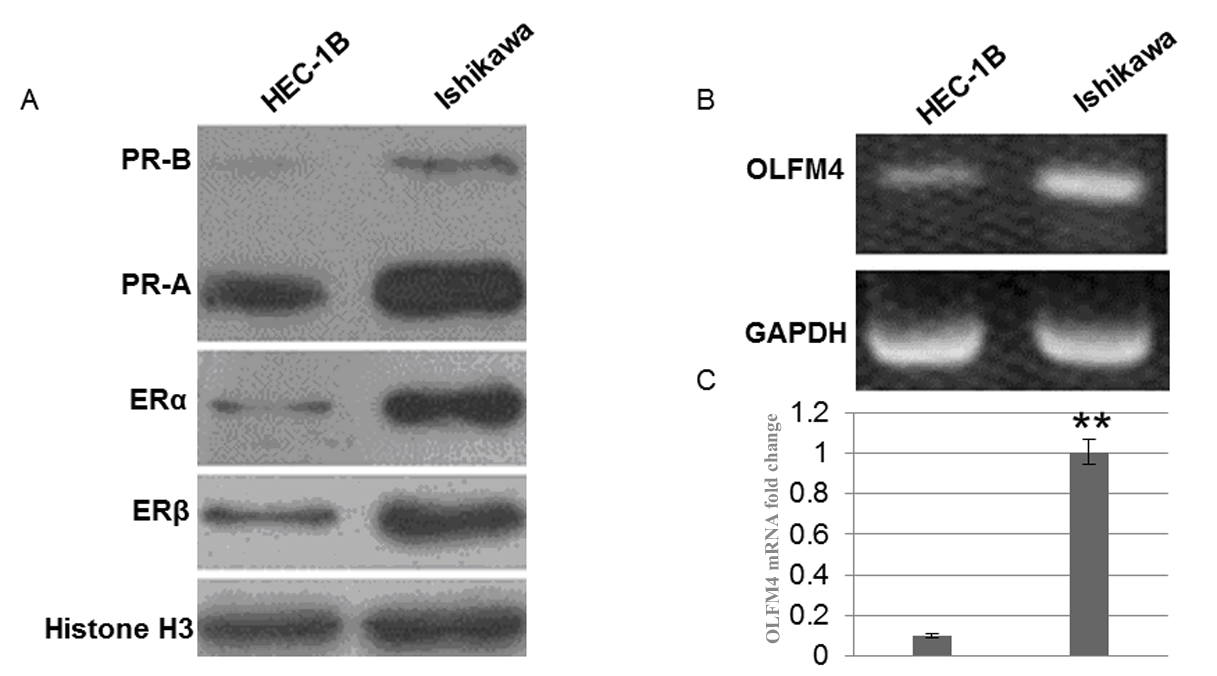

Supplement: Supplementary file 1 — Figure S1. Expression of OLFM4, ER and PR in endometrial carcinoma cells. [file jcmm0018-0863-SD1.tif]

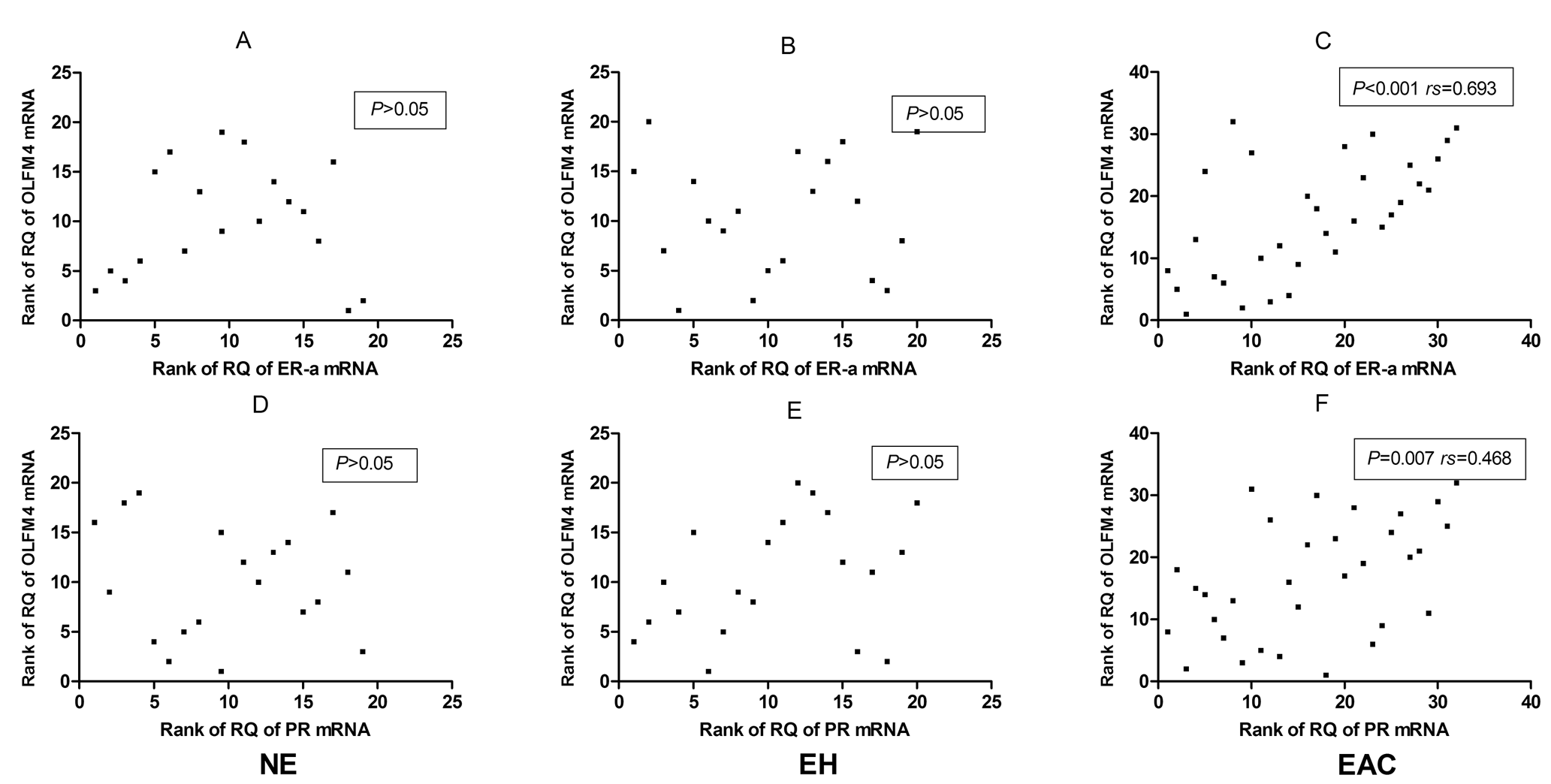

Supplement: Supplementary file 2 — Figure S2. Co-relationships of OLFM4 with ERα or PR in mRNA level in normal endometrium and endometrial lesions. [file jcmm0018-0863-SD2.tif]
