# Supplementary material for: Oestrogen receptor-mediated expression of Olfactomedin 4 regulates the progression of endometrial adenocarcinoma
Source: J Cell Mol Med. 2014 Feb 4;18(5):863–74. doi: 10.1111/jcmm.12232 (PMC4119392; doi:10.1111/jcmm.12232)
Supplement: Supplementary file 3 — Table S1. UniGene libraries involved in digital differential display. [file jcmm0018-0863-SD3.doc]

Supplementary Table S1. UniGene libraries involved in digital differential display

| Libraries | Libraries ID | ESTs | Putative genes |
| --- | --- | --- | --- |
| Well-differentiated endometrial adenocarcinoma | 1461 | 20,758 | 8,688 |
| Moderately-differentiated endometrial adenocarcinoma | 1462 | 13,238 | 6,351 |
| Poorly-differentiated endometrial adenocarcinoma | 1463 | 8,338 | 4,434 |
