# Supplementary material for: Oestrogen receptor-mediated expression of Olfactomedin 4 regulates the progression of endometrial adenocarcinoma
Source: J Cell Mol Med. 2014 Feb 4;18(5):863–74. doi: 10.1111/jcmm.12232 (PMC4119392; doi:10.1111/jcmm.12232)
Supplement: Supplementary file 4 — Table S2. Primers used for PCR analysis in the study. [file jcmm0018-0863-SD4.doc]

Supplementary Table S2. Primers used for PCR analysis in the study

| Genes | Primer sequences | Product size (bp) |
| --- | --- | --- |
| OLFM4-F | aaggagagttttggtggaagc | 255 |
| OLFM4-R | agacggtttgctgatgttcac |
| ERα-F | ggtgccctactacctggagaa | 144 |
| ERα-R | gccatacttcccttgtcattg |
| PR-F | acacctccagttctttgctgac | 258 |
| PR-R | attctttcatccgctgttcatt |
| GAPDH-F | agaaggctggggctcatttg | 249 |
| GAPDH-R | aggggccatccacagtcttc |
