# Supplementary material for: Oestrogen receptor-mediated expression of Olfactomedin 4 regulates the progression of endometrial adenocarcinoma
Source: J Cell Mol Med. 2014 Feb 4;18(5):863–74. doi: 10.1111/jcmm.12232 (PMC4119392; doi:10.1111/jcmm.12232)
Supplement: Supplementary file 5 — Table S3. Expression profile of OLFM4 analysed by EST count. [file jcmm0018-0863-SD5.doc]

Supplementary Table S3. Expression profile of OLFM4 analyzed by EST count

| Tissue pools | Expression level | |
| --- | --- | --- |
|  | Transcripts per million (TPM) | Gene EST/total EST in pool |
| ascites | 25 | 1/39834 |
| bladder | 2645 | 79/29860 |
| brain | 1 | 2/1092688 |
| breast | 33 | 5/151230 |
| cervix | 20 | 1/48486 |
| intestine | 758 | 176/231981 |
| kidney | 180 | 38/210778 |
| mouth | 30 | 2/66150 |
| muscle | 75 | 8/106371 |
| nerve | 128 | 2/15535 |
| pancreas | 103 | 22/213440 |
| pharynx | 24 | 1/40725 |
| prostate | 100 | 19/189536 |
| stomach | 114 | 11/95679 |
| testis | 2 | 1/435204 |
| thymus | 87 | 7/79697 |
| **uterus** | **236** | **55/232093** |
| breast tumor | 42 | 4/93090 |
| colorectal tumor | 622 | 70/112517 |
| gastrointestinal tumor | 84 | 10/118498 |
| germ cell tumor | 11 | 3/263230 |
| head and neck tumor | 14 | 2/133826 |
| pancreatic tumor | 114 | 12/105004 |
| **uterine tumor** | **577** | **52/90107** |

Note: The pools of libraries in which OLFM4 transcripts were not observed were excluded in the table.
