# Supplementary material for: Oestrogen receptor-mediated expression of Olfactomedin 4 regulates the progression of endometrial adenocarcinoma
Source: J Cell Mol Med. 2014 Feb 4;18(5):863–74. doi: 10.1111/jcmm.12232 (PMC4119392; doi:10.1111/jcmm.12232)
Supplement: Supplementary file 6 — Table S4. Expression of OLFM4, ERα and PR in endometrial tissues detected by IHC. [file jcmm0018-0863-SD6.doc]

Supplementary Table S4. Expression of OLFM4, ERα, and PR in endometrial tissues detected by IHC

| Tissues | OLFM4 (cases/%) | |  | ERα (cases/%) | |  | PR (cases/%) | |
| --- | --- | --- | --- | --- | --- | --- | --- | --- |
|  | High-exp | Low-exp |  | High-exp | Low-exp |  | High-exp | Low-exp |
| Endometrium^∏^ | 13/43.3 | 17/56.7 |  | 29/96.7 | 1/3.3 |  | 29/96.7 | 1/3.3 |
| Hyperplasia^§^ | 18/60.0 | 12/40.0 |  | 28/93.3 | 2/6.7 |  | 29/96.7 | 1/3.3 |
| Atypical hyperplasia^‖^ | 20/66.7 | 10/33.3 |  | 22/73.3 | 8/26.7 |  | 25/83.3 | 5/16.7 |
| Adenocarcinoma^∮^ | 136/68.0 | 64/32.0 |  | 91/45.5 | 109/54.5 |  | 133/66.5 | 67/33.5 |
| *P* | 0.064 | |  | <0.001 | |  | 0.001 | |

Bonferroni test was used for multiple comparisons between groups the significance level was adjusted to 0.05/6=0.0083.

Multiple comparisons for OLFM4: ^∏—§^*P*=0.196, ^∏—‖^*P*=0.069, ^∏—∮^*P*=0.008, ^§—‖^*P*=0.592, ^§—∮^*P*=0.385, ^‖—∮^*P*=0.884.

Multiple comparisons for ERα: ^∏—§^*P*=1.000, ^∏—‖^*P*=0.026, ^∏—∮^*P*<0.001, ^§—‖^*P*=0.038, ^§—∮^*P*<0.001, ^‖—∮^*P*=0.004.

Multiple comparisons for PR: ^∏—§^*P*=1.000, ^∏—‖^*P*=0.195, ^∏—∮^*P*=0.001, ^§—‖^*P*=0.195, ^§—∮^*P*=0.001, ^‖—∮^*P*=0.064.
