# Supplementary material for: Oestrogen receptor-mediated expression of Olfactomedin 4 regulates the progression of endometrial adenocarcinoma
Source: J Cell Mol Med. 2014 Feb 4;18(5):863–74. doi: 10.1111/jcmm.12232 (PMC4119392; doi:10.1111/jcmm.12232)
Supplement: Supplementary file 7 — Table S5. Differentiation of endometrioid adenocarcinoma and OLFM4 expression level. [file jcmm0018-0863-SD7.doc]

Supplementary Table S5. Differentiation of endometrioid adenocarcinoma and OLFM4 expression level

| Level of differentiation | OLFM4 | | *P* |
| --- | --- | --- | --- |
|  | High-exp (cases/%) | Low-exp (cases/%) |  |
| Well-differentiated^∏^（n=86） | 70/81.4 | 16/18.6 | <0.001 |
| Moderately-differentiated^§^（n=84） | 55/65.5 | 29/34.5 |  |
| Poorly-differentiated**^‖^**（n=30） | 11/36.7 | 19/63.3 |  |

^∏—§^*P*=0.019, ^§—^**^‖^***P*=0.006, ^∏—^**^‖^***P*<0.001. For multiple comparisons between groups the significance level was adjusted to 0.05/3=0.017.
